# Crosslinking with transglutaminase does not change metabolic effects of sodium caseinate in model beverage in healthy young individuals

**DOI:** 10.1186/1475-2891-11-35

**Published:** 2012-06-01

**Authors:** Kristiina R Juvonen, Martina E Lille, David E Laaksonen, Hannu M Mykkänen, Leo K Niskanen, Karl-Heinz Herzig, Kaisa S Poutanen, Leila J Karhunen

**Affiliations:** 1 Food and Health Research Centre, Department of Clinical Nutrition, Institute of Public Health and Clinical Nutrition, University of Eastern Finland, P.O. Box 1627, 70211, Kuopio, Finland; 2 VTT Technical Research Centre of Finland, 02150, Espoo, Finland; 3 Department of Medicine, Kuopio University Hospital, 70211, Kuopio, Finland; 4 Institute of Biomedicine, Physiology, University of Eastern Finland, 70211, Kuopio, Finland; 5 Department of Medicine, Central Hospital Central Finland, 70460, Jyväskylä, Finland; 6 University of Eastern Finland, 70211, Kuopio, Finland; 7 Department of Psychiatry, Kuopio University Hospital, 70211, Kuopio, Finland; 8 Institute of Biomedicine, Division of Physiology and Biocenter of Oulu, Oulu University, 90014, Oulu, Finland

**Keywords:** Caseinate, Whey protein, Crosslinking, Postprandial, Glucose, Insulin, Amino acids, Appetite

## Abstract

**Background:**

Postprandial metabolic and appetitive responses of proteins are dependent on protein source and processing technique prior to ingestion. Studies on the postprandial effects of enzymatic crosslinking of milk proteins are sparse. Our aim was to study the effect of transglutaminase (TG)-induced crosslinking of sodium caseinate on postprandial metabolic and appetite responses. Whey protein was included as reference protein.

**Methods:**

Thirteen healthy individuals (23.3 ± 1.1 y, BMI 21.7 ± 0.4 kg/m^2^) participated in a single-blind crossover design experiment in which the subjects consumed three different isovolumic (500 g) pourable beverages containing either sodium caseinate (Cas, 29 g), TG-treated sodium caseinate (Cas-TG, 29 g) or whey protein (Wh, 30 g) in a randomized order. Blood samples were collected at baseline and for 4 h postprandially for the determination of plasma glucose, insulin and amino acid (AA) concentrations. Gastric emptying (GE) was measured using the ^13^ C-breath test method. Appetite was assessed using visual analogue scales.

**Results:**

All examined postprandial responses were comparable with Cas and Cas-TG. The protein type used in the beverages was reflected as differences in plasma AA concentrations between Wh and Cas, but there were no differences in plasma glucose or insulin responses. A tendency for faster GE rate after Wh was detected. Appetite ratings or subsequent energy intake did not differ among the protein beverages.

**Conclusions:**

Our results indicate that the metabolic responses of enzymatically crosslinked and native sodium caseinate in a liquid matrix are comparable, suggesting similar digestion and absorption rates and first pass metabolism despite the structural modification of Cas-TG.

## Background

Food digestion is affected by the characteristics of macronutrients as well as by the micro- and macrostructure of the food, which may have pronounced effects on subsequent metabolic and appetitive responses [1-6]. Despite the progress in our understanding of the regulation of postprandial metabolism and energy homeostasis, attempts to influence physiological mechanisms and energy balance by altering the structural characteristics of foods have been modest.

Modification with crosslinking enzymes such as transglutaminase (TG) has been used extensively to change the functionality of proteins and thereby to improve the textural quality and stability of protein-based food products [7]. In dairy products, TG-induced crosslinking can increase the firmness and water-holding capacity of acid-induced gels in products with low solids and fat content or to improve the stability of emulsions and foams [8]. Despite the evidence on the benefits of TG-induced crosslinking in milk protein-based products, we are still a long way from fully understanding its effects on protein digestion in the gastrointestinal (GI) tract. Some *in vitro* digestion studies suggest that crosslinking with TG renders casein more resistant to proteolysis by pepsin and trypsin [9,10], but the results have not been confirmed in humans. Since TG action results in crosslinks between glutamine and lysine residues in proteins, some concern has been raised over the bioavailability of lysine in TG-treated products. In animal studies (rats), however, the isopeptide bond formed by TG between glutamine and lysine residues appears to be cleaved by enzymes present in the GI tract or kidneys [11,12]. Roos et al. [13] reported that crosslinking of casein with TG does not inhibit proteolysis *in vitro* or digestibility in the intestinal tract of miniature pigs when fed as part of a mixed diet.

Whey protein has been described as a “fast” soluble protein after which gastric emptying (GE) is rapid and the postingestive amino acid (AA) response high, fast and transient. In contrast, the “slow” casein protein precipitates at the low pH prevailing in the stomach, resulting in a delayed GE rate and a lower, slower and prolonged AA response [14,15]. However, TG-crosslinking may change casein’s clotting behaviour in the stomach. Flanangan et al. [16] showed that the solubility of TG-treated sodium caseinate in water is improved at pH-values between 2 and 6, which might result in a higher digestion and GE rate. On the other hand, crosslinking with TG results in stronger gels when a sodium caseinate solution is slowly acidified to pH 4.6 [17], which might delay digestion and GE. Also other factors than pH, such as meal dilution [18] and components either from the food or gastric juice need to be taken into account when considering the behaviour of proteins in the stomach. NaCl, for instance, is known to limit the solubility of TG-treated sodium caseinate to a great extent at pH 3.2 [19].

We recently found that TG-induced crosslinking of sodium caseinate modified both postprandial appetite and metabolic responses [20] after a test meal with a rather high protein concentration (13%), where the TG treatment changed the form of the test product from a high-viscous liquid to a strong gel. The altered responses might thus not only be the result of the crosslinking *per se,* but the difference in the form of the test products might also play an important role. Therefore, to better understand the postmeal effects of protein crosslinking, we investigated the effect of TG-induced crosslinking of sodium caseinate on postprandial metabolic and appetitive responses in healthy young individuals using test products with less pronounced differences in rheological properties. By lowering the protein content to 6%, pourable model beverages could be prepared both with and without TG treatment. Non-crosslinked whey protein was included as reference protein. We hypothesized that crosslinking of sodium caseinate could alter its clotting behaviour in the stomach and thereby affect the rates of digestion and gastric emptying, which could result in changes in subsequent postprandial responses of plasma glucose, insulin and amino acids as well as appetite ratings.

## Methods

### Subjects

A total of 13 healthy normal-weight individuals (11 female, 2 male) were recruited via an intranet announcement to participate in a study at the Department of Clinical Nutrition at the University of Eastern Finland (Table 1).

**Table 1:** Participant characteristics^*^

**Characteristic**	**Value**	**SEM**
Age (years)	23.3	1.1
Weight (kg)	62.8	2.3
Height (m)	1.70	0.02
Body mass index (kg/m^2^)	21.7	0.4
Systolic blood pressure (mm Hg)	116.0	2.9
Diastolic blood pressure (mm Hg)	72.2	1.9
Oral-glucose-tolerance		
Plasma glucose, 0 min (mmol/l)	5.3	0.1
Plasma glucose, 120 min (mmol/l)	5.2	0.3
Three-Factor Eating Questionnaire		
- cognitive restraint of eating (factor 1)	8.9	1.2
- disinhibition (factor 2)	4.8	0.6
- hunger (factor 3)	4.1	0.6

^*^Mean values with their standard errors, *n* 13 (11 female, 2 male).

During the screening phase volunteers were interviewed about their medical history, dietary habits and physical activity. Individuals were excluded if they had any allergies or food intolerances, did not eat breakfast, had modified their diet or exercise patterns during the past year to lose weight, were taking medication (except oral contraceptives) or were smokers. The Three-Factor Eating Questionnaire (TFEQ) [21] was used to exclude subjects with abnormal eating behavior. All participants had normal glucose tolerance (fasting plasma glucose ≤5.7 mmol/l and 2-h glucose <7.8 mmol/l) determined by the oral glucose tolerance test (OGTT). Participants were individually familiarized with the study procedures and measurements prior to the actual study visits to reduce potential confounding factors due to misunderstanding.

This study was conducted according to the guidelines laid down in the Declaration of Helsinki and all procedures involving human subjects were approved by the Ethics Committee of the University of Kuopio and Kuopio University Hospital. Written informed consent was obtained from all subjects.

### Preparation of sodium caseinate powders for test products

A batch of TG-treated sodium caseinate powder was prepared for the study. A non-enzyme-treated reference material was prepared as well. Sodium caseinate (EM7) from DMW International (The Netherlands), composed of 90% protein, 5% moisture, 4% ash, 0.8% fat and 0.2% lactose, was used as the casein source. TG powder (Activa MP) from Ajinomoto Foods Europe SAS (France), consisting of 94% maltodextrin + lactose, 5% moisture and 1% protein, was used as the crosslinking enzyme. The TG activity of the powder was determined using 0.03 M N-carbobenzoxy-L-glutaminyl-glycine (Sigma) as the substrate at pH 6.0 according to Folk [22]. For the TG powder an activity of 2600 nkat/g was measured. A single nanokatal (nkat) is defined as the amount of enzyme activity that converts 1 nmol/s of the substrate used in the assay conditions.

Powders were prepared by mixing 1169 g sodium caseinate with 9000 g cold tap water in a Diosna spiral mixer (SP 24 F/E, Dierks & Söhne GmbH, Germany). After initial mixing, 8000 g of water heated to 90 °C was added, and mixing continued until a homogeneous solution was obtained. The protein content of the solution was approximately 5.5% at this point. The sodium caseinate solution was poured into a 40 litre fermenter (IF 40, New Brunswick Scientific, USA) and warmed up to 40 °C under gentle stirring (50 rpm). A 10% suspension of TG in water was prepared and 754 g of the suspension was added to the sodium caseinate solution, which corresponds to 200 nkat TG per g sodium caseinate. The sodium caseinate content of the mixture was adjusted to 5% by adding the required amount of water. The mixture was incubated for 2 h at 40 °C for the transglutaminase reaction and then heated at 90 °C for 15 min to inactivate the enzyme, cooled, frozen at −20 °C and freeze-dried. The non-enzyme-treated reference powder was prepared according to the same protocol, but the TG suspension was heated for 5 min in a boiling water bath (to inactivate the enzyme) prior to adding it to the sodium caseinate solution. The microbiological quality of the test powders fulfilled the regulatory requirements set for milk powders.

### Test products

A total of three isoenergetic and isovolumic dairy protein based beverages containing a) untreated sodium caseinate (Cas), b) transglutaminase-treated sodium caseinate (Cas-TG) and c) untreated whey protein (Wh), were served as test products. The used ingredients and detailed composition of the test products are shown in Tables 2 and 3, respectively. Whey protein was included as a reference product due to the previously demonstrated distinct postprandial effects of whey protein as compared to casein [14,23,24]. Furthermore, beverage portions contained 40 g of glucose to stimulate glucose-dependent postprandial responses and to mimic the nutritional composition of a mixed diet.

**Table 2:** Ingredients used in the test beverages (g/portion)

	**Cas**	**Cas-TG**	**Wh**
Protein powder (g)	33.15^*^	34.05^†^	32.26^‡^
Glucose (g)^§^	40.0	40.0	40.0
Aroma (g)^║^	4.0	4.0	4.0
^13^ C acetic acid (mg)^¶^	50	50	50
Water up to 500 g			

Cas, caseinate; Cas-TG, transglutaminase treated Cas; Wh, whey protein.

^*^Non-enzyme-treated sodium caseinate powder prepared for the study, composition: 86.3% protein from sodium caseinate, 6.1% maltodextrin + lactose (from TG and sodium caseinate powder), 3.8% ash, 2.9% moisture, 0.8% fat, 0.06% TG-enzyme (inactivated), DMV International, Veghel, The Netherlands.

^†^TG-treated sodium caseinate powder prepared for the study, composition: 84.0% sodium caseinate, 5.0% maltodextrin + lactose (from TG powder), 5.5% moisture, 3.7% ash, 0.8% fat, 0.06% TG-enzyme.

^‡^Whey protein isolate (mainly beta-lactoglobulin), *Bi*PRO®, composition: 92.9% protein, 5% moisture, 1.9% ash, 0.4% fat and 0.5% lactose, Davisco Foods International, Inc., USA.

^§^Anhydrous glucose, composition 100% dextrose anhydre CF, moisture 0.5%, Oriola, Espoo, Finland.

^║^Orange aroma, Spice Aroma Finland Ky, Kuopio, Finland.

^¶13^ C labeled acetic acid for measuring gastric emptying, Wagner Analysen Technik GmbH, Bremen, Germany.

**Table 3:** Composition of test beverages^*^

	**Cas**	**Cas-TG**	**Wh**
Portion size (g)	500	500	500
Energy (kJ (kcal))	1194 (281)	1172 (276)	1163 (274)
Energy density (kJ/g)	2.39	2.34	2.33
Protein (g (E%^†^))	28.6 (41)	28.6 (41)	30.0 (44)
Carbohydrates (g (E%^†^))	41.0 (58)	39.7 (58)	38.2 (56)
Fat (g (E%^†^))	0.3 (1)	0.3 (1)	0.1 (0)

Cas, caseinate; Cas-TG, transglutaminase treated Cas; Wh, whey protein.

^†^Percentages of total energy content.

^*^Ingested with 100 ml of water.

The test beverages were prepared by dissolving the anhydrous glucose powder in water. Then the protein powder was added to the glucose solution and stirred until completely dissolved. The test beverages were stored in the refrigerator (5 °C) overnight covered with plastic film. In the morning of the test day the volume of the test beverages was leveled up to 500 g with water, and a natural orange aroma and ^13^ C acetic acid as a marker for gastric emptying were added to each test beverage just before serving.

### Characterization of the test products

**Figure 1:**
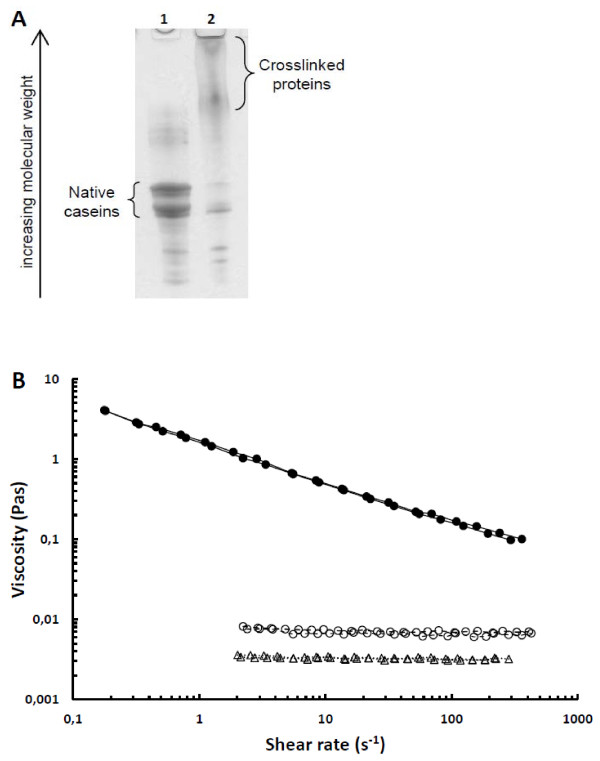
**(A) SDS-PAGE-analysis of sodium caseinate powders prepared for the study.** Lane 1) non-enzyme-treated sodium caseinate, lane 2) sodium caseinate crosslinked with transglutaminase. (**B**) Viscosity of test products as function of shear rate. For each sample, the results of two replicate measurements are shown, caseinate (−−○−−), transglutaminase-treated caseinate (−●–) or whey protein (··▽··).

The level of protein crosslinking of the sodium caseinate powders was analysed by sodium dodecyl sulfate polyacrylamide gel electrophoresis (SDS-PAGE) as described by Ercili Cura et al. [17]. The TG-treated powder was, as shown in Figure 1A, extensively crosslinked in comparison with the non-enzyme-treated powder. Ercili Cura et al. [17] showed that even a lower level of sodium caseinate crosslinking resulted in marked changes in rheological properties and microstructure of gels produced by acidification to pH 4.6.

The viscosity of the test products was measured at 20 °C with a stress controlled rotational rheometer (AR-G2, TA Instruments, UK) equipped with a concentric cylinder geometry (diameter of cup and bob 30 and 28 mm, respectively). After loading the samples to the measuring geometry, they were allowed to rest for 5 min before the measurement was started. The steady-state viscosity of the samples was measured in duplicate with a gradually increasing shear stress with values resulting in shear rates in the range 0.1-500 s^-1^.

The test product prepared from the crosslinked sodium caseinate powder showed a higher viscosity than the ones prepared from non-crossliked sodium caseinate or whey protein (Figure 1B), but all test products still remained pourable, in contrast to our previous study [20], in which crosslinking with TG resulted in the formation of a very rigid gel.

### Study design

Each subject participated in the study with a single-blind, randomized, crossover design where all participants tested each beverage with more than 2 d between the tests. The subjects were instructed to maintain their habitual diet and exercise routines as constant as possible, refrain from heavy exercise on the day before the study visit and avoid alcohol consumption for 2 d before the test day. Subjects were requested to arrive to each laboratory visit the same way (e.g. walking, cycling, car or bus) and to avoid extra physical stress before the beginning of the visit. At the beginning of each experiment the participants were weighed and their alcohol consumption and the amount of exercise during the previous day were checked by an interview.

All the study visits began in the morning after a 10–12 h fast. During the first study visit oral glucose tolerance test (OGTT, 75 g glucose dissolved in 300 ml water), was performed to ascertain normal glucose tolerance. The OGTT procedure had otherwise exactly the same study protocol as the following three study sessions to familiarize the subjects with the study protocol and measures. During the actual study visits, subjects consumed one of the chilled test beverages between 07.45–08.15 hours along with 100 ml of tap water in a randomized order: a beverage containing either (1) untreated sodium caseinate, (2) transglutaminase (TG) treated sodium caseinate or (3) whey protein isolate (Table 2 and 3).

Blood samples were taken for the determination of plasma glucose, insulin, and amino acid concentrations via an indwelling cannula placed in the forearm 10 min prior to the first blood sampling. Venous samples were collected at baseline and 15, 30, 45, 60, 90, 120, 180 and 240 min after the ingestion of the test beverages. Subjects rated their appetite at the concomitant time points immediately after the blood sampling in a separate room. Breath test samples were collected immediately before blood sampling at baseline and 15, 30, 45, 60, 75, 90, 105, 120, 150, 180, 210, 240 min after the test beverage consumption. Subjects were requested to avoid any extra walking and stay in sitting position during the study period to minimize the effects of body posture on postprandial responses.

### Appetite measurements

Appetite profile (hunger, satiety, desire to eat, fullness, thirstiness), and pleasantness of the test meals were evaluated using Visual Analogue Scales (VAS). Each VAS scale had 100 mm horizontal line with verbal anchors (in Finnish) at either end expressing the weakest or strongest statement (i.e. ‘I am not hungry at all’ or ‘I have never been more hungry’). The participants were advised to draw a vertical line on the horizontal axis corresponding to their sensations at the time of assessment. VAS ratings were measured in millimeters, resulting in scores between 0 and 100 for statistical analyses.

### Food intake

Study participants were advised to keep detailed 24 h food records to monitor their food intake during the study. These included food records two days before each study day, half-day record for the remaining day of each experiment and the following day to reflect the baseline diet and the effects of each test product on subsequent food intake. The average daily energy and macronutrient intake of the participants from the food records were analyzed by using the MICRO-NUTRICA database (version 2.5; Finnish Social Insurance Institution, Turku, Finland).

### Biochemical measurements

Plasma samples for measurements of insulin and AA were collected in prechilled EDTA-containing tubes and fluoride citrate-containing tubes were used for plasma glucose. Plasma insulin and AA samples were centrifuged within 15 min, for 15 min at 1700 x g at 4°C. Plasma glucose samples were centrifuged for 10 min at 2400 x g at 4°C. All samples were immediately stored in −70°C until analysed. Results were obtained from all the study participants except for plasma AA measurements for which 10 randomly selected individuals were included.

Plasma glucose was analysed using an enzymatic photometric assay (Konelab 20XTi Clinical Chemistry Analyzer, Thermo Electron Corp, Vantaa, Finland) and plasma insulin using a luminometric immunoassay (ADVIA Centaur Immunoassay System, Siemens Medical Solutions Diagnostics, Tarrytown, NY, USA). The intra-assay CV for the plasma glucose was 2.7% at 10.2 mmol/l and the inter-assay CV was 4.1% at 2.05 mmol/l and 1.8% at 8.2 mmol/l. For plasma insulin, the intra-assay CV was 2.7% at 667 pmol/l and the inter-assay CV was 6.6% at 41 pmol/l and 5.1% at 444 pmol/l.

Plasma amino acid concentrations were analysed with a Mass TRAK^TM^ Amino Acid Analysis Application Solution (Waters). AccQ·Fluor reagent kit, Mass TRAK^TM^ Amino Acid Analysis concentrate A and eluent B were obtained from Waters (Milford, MA, USA). Amino Acid Standard Solution, Amino Acid Standards Physiological, Basics, L-isoleusine and glutamine were obtained from Sigma-Aldrich (St. Luis, Missouri, USA). A total volume of 50 μl of sample was mixed with 200 μl of water and 50 μl of norvaline (0.025 mM) was added as an interval standard. 300 μl of acetonitrile was added and after vortex mixing the sample was filtrated through a sirocco plate (Waters lot no 828001). Next, the extracts were freeze-dried and reconstituted with 50 μl of water. Derivatization was done with an AccQ·Fluor reagent kit**.** AccQ·Fluor reagent was reconstituted with acetonitrile (350 μl), vortexed for 10 seconds, heated at 55°C and vortex until dissolved. AccQ·Fluor reagent (20 μl) was added to 10 μl of the extract and 70 μl of boric acid buffer, and the sample mixture instantly vortexed for 60 seconds. Samples were kept at 5 °C before and during analysis. Analysis was performed on an Acquity UPLC system (Waters, USA) with a diode array detector. Chromatography was performed using an Acquity Mass Trak^TM^ column (2.1 x 150 mm, 1.7 μm, (Waters Corporation, USA) kept at 43°C. The injection volume was 1.0 μl. Separation was performed using gradient elution with 10% (v/v) Amino Acid Analysis Concentrate A in water (A) and Amino Acid Analysis Eluent B (B) at a flow rate of 0.4 ml/min. Signal was detected at 260 nm (2.4 nm resolution, 20 points/second).

In addition to single amino acids, branched-chain (BCAA; valine, isoleucine, leucine), essential (EAA; leucine, isoleucine, valine, lysine/tyrosine (lysine and tyrosine concentrations counted up), phenylalanine, threonine, tryptophan, arginine, histidine, cysteine) and total plasma amino acid (TAA; glysine, alanine valine, leucine, isoleucine, praline, phenylalanine, tryptophan, serine, threonine, cysteine, methionine, arginine, histidine, lysine/tyrosine, aspartic acid, glutamic acid, asparagines, glutamine, ornithine, alpha amino-butyric acid, citrulline, sarcosine, ethanolamine, carnosine, taurine, 1-methyl-l-histidine, 3-methyl-l-histidine) concentrations were summarized and included in the analyses.

### Gastric emptying

Gastric emptying was assessed using the standardized ^13^ C-Acetate Breath Test -method in combination with the quantitative isotope gas mixture analyser, IRIS ^13^ C-Breath Test System (IRIS, non-dispersive infrared spectroscopy; Wagner Analysen Technik GmbH, Bremen, Germany). GE is estimated by monitoring the appearance of ^13^CO_2_ in breath test samples subsequent to consumption and metabolism of ^13^ C-acetate labeled test product adjusted by body weight and height of individual participants. Analysis of the ^13^CO_2_ appearance in the samples provides estimates of individual GE parameters, as described by the time with maximum speed of GE after ingestion the test meal (t_lag_), the time when the first half of the ^13^ C-labelled substrate dose of the test meal has been metabolised (t_1/2_) and the GE coefficient (GEC), which is a measure of initial gradient of GE.

### Statistical methods

The data analyses were performed with SPSS for Windows software (SPSS for WINDOWS, version 17.0, USA). The results are expressed as mean and standard error of the mean (SEM) with a value *P* ≤ 0.05 (2-sided) as a criterion for the statistical significance.

Linear mixed-effects modelling was used to compare the effects of the test meals on the postprandial metabolic and appetite responses. In the analysis, the baseline value of each parameter was used as a covariate to take into account the possible effect of baseline differences on the analysis. The method takes into account the sources of variation where the subject is used as a random factor and product, time and product x time as fixed factors. Where a significant main effect of a product, time or product x time interaction was observed (*P* < 0.05), post-hoc analyses were performed using the Bonferroni correction for multiple comparisons.

## Results

### Plasma glucose and insulin responses

**Figure 2:**
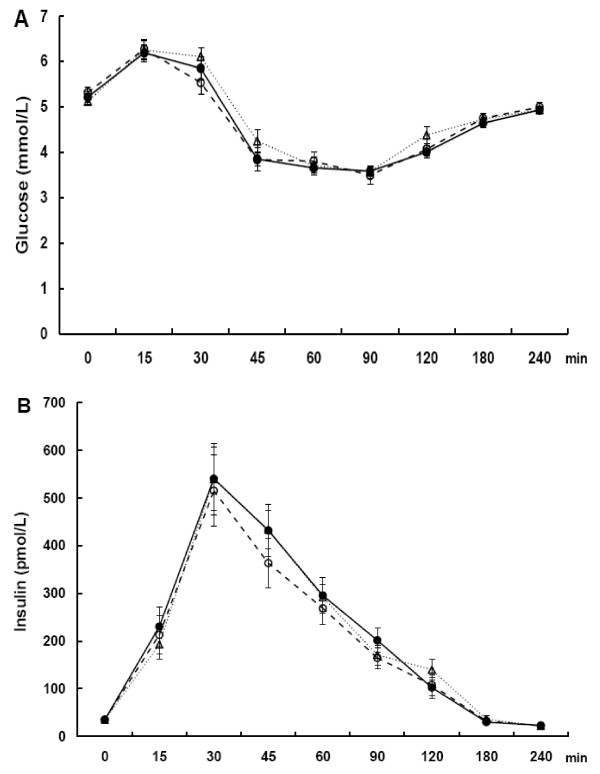
**Changes in the concentration of plasma glucose (A) and plasma insulin (B) during the 240 min postprandial period in young individuals consuming beverages composed of caseinate (−−○−−), transglutaminase-treated caseinate (−●–) or whey protein (··▽··).** Values are means ± SEM, *n* = 13.

After all test beverages plasma glucose responses increased initially from baseline up to 15 min, declined thereafter towards the nadir at 90 min and returned close to the baseline at 240 min (Figure 2A). Postmeal insulin responses increased similarly after all test beverages and peaked at 30 min after which the concentrations returned towards the baseline (Figure 2B). No significant differences were detected in postmeal glucose and insulin responses between Cas and Cas-TG or between Cas/Cas-TG and Wh beverages (*P* > 0.05).

### Plasma amino acid responses

**Figure 3:**
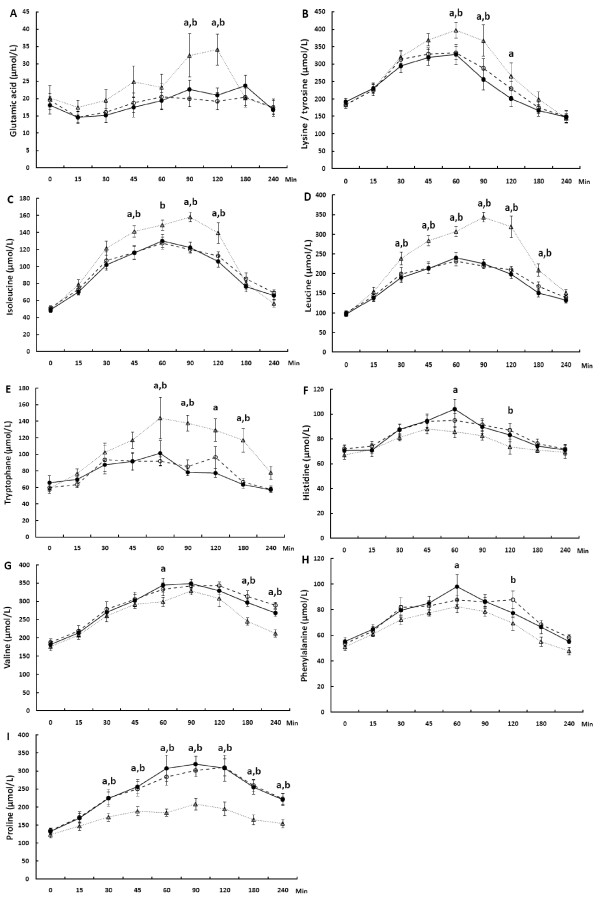
**Changes in the concentration of plasma glutamic acid (A), lysine/tyrosine (B), isoleucine (C), leucine (D), tryptophan (E), histidine (F), valine (G), phenylalanine (H) and proline (I) during the 240 min postprandial period in young individuals consuming beverages composed of caseinate (−−○−−), transglutaminase-treated caseinate (−●–) or whey protein (··▽··).** Values are means ± SEM, n = 10, a) whey protein different from Cas-TG (*P*<0.05), b) whey protein different from Cas (*P*<0.05).

**Figure 4:**
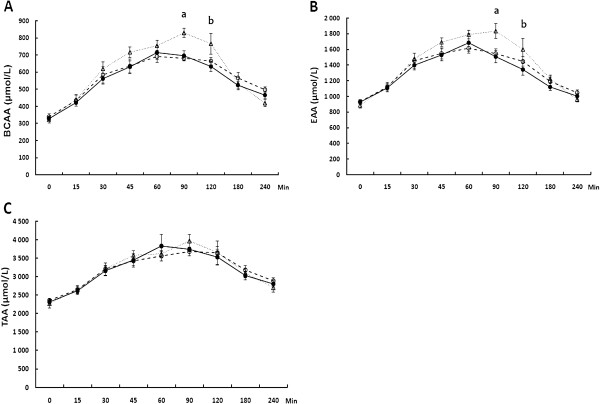
**Changes in the concentration of plasma branched-chain amino acids (BCAA) (A), essential amino acids (EAA) (B), and total amino acids (TAA) (C) during the 240 min postprandial period in young individuals consuming beverages composed of caseinate (−−○−−), transglutaminase-treated caseinate (−●–) or whey protein (··▽··).** Values are means ± SEM, n = 10, a) whey protein different from Cas and Cas-TG (*P* < 0.05), b) whey protein different from Cas-TG (*P* < 0.05).

Plasma amino acid profiles with significant differences among the test products are shown in Figures 3 and 4.

No significant differences were detected between Cas and Cas-TG in any of the postprandial amino acid concentrations. The concentrations of glutamic acid, lysine/tyrosine, isoleucine, leucine and tryptophan were increased more after whey protein than after Cas or Cas-TG (Figure 3A–E). On the other hand, postprandial concentrations of valine, histidine, phenylalanine and proline were lower after whey protein than after Cas or Cas-TG (Figure 3F–I).

Postprandial BCAA and EAA concentrations were comparable between Cas and Cas-TG (*P* > 0.05). After Wh BCAA and EAA concentrations were significantly increased at 90 min when compared to Cas and Cas-TG (*P* < 0.05) and at 120 min when compared to Cas-TG (*P* < 0.05) (Figure 4A–C). No significant differences were detected postprandially among the beverages in total AA concentrations.

### Gastric emptying

There were no significant differences in gastric half emptying time (t_1/2_) and gastric emptying coefficient (G.E.C.) among the test beverages (*P* > 0.05). The time with maximum speed of GE after ingestion the test meal (t_lag_) tended to differ between Cas and Wh so that the time was greater for Cas than for Wh (*P* = 0.061) while no difference was observed between Cas and Cas-TG.

### Test product consumption and appetite ratings

The mean time for the consumption of the test products was comparable: 2.2 (SEM 0.2) min for Cas, 2.1 (SEM 0.2) min for Cas-TG, and 2.2 (SEM 0.1) min for Wh.

All appetite responses varied significantly during the experimental period (*P* < 0.05) (data not shown). Hunger, desire to eat and thirst reached nadir at 15–30 min and returned towards the baseline thereafter. Feelings of satiety and fullness peaked at 15 min and gradually declined towards the baseline values during the last hours of the study period. No significant differences were detected in any of the appetite responses among the test products.

### Food consumption

Food consumption monitored by food diaries indicated that energy intake and carbohydrate, fat and dietary fibre consumption were comparable among the days before each study day, the remaining day and the following day. Dietary protein intake was greater after Cas-TG when compared to Cas for the remaining of the study day (84.9 vs. 65.5 g, *P* < 0.05). On the other days protein intake was comparable among the test products.

## Discussion

We found that in healthy young women and men, postprandial metabolic and appetitive responses were comparable between the structurally modified and native caseinate when consumed in the beverage form. This was in contrast to our hypothesis that enzymatic crosslinking of caseinate with TG would slow the GE rate with subsequent alterations in the postprandial responses of AA, glucose and insulin. In line with earlier observations [23,25], we found that Cas and Wh have protein-specific effects on postprandial AA concentrations. Unlike some previous reports [24,25], the different AA profiles were not reflected in differences in postprandial responses related to insulin and glucose metabolism or appetite responses. This might partly be explained by the lower protein dosage, concentration and/or the carbohydrate source used in our study. In our previous study [20], caseinate protein was modified by intensive TG treatment, which induced gel formation due to the high protein content (13%) of the test product. Consequently both the molecular structure and the physical form of the test product were changed. The effects of this protein modification were reflected in the postprandial appetite and metabolic responses so that after the TG-treated solid caseinate test product fullness was increased and glucose, insulin, glucagon-like peptide 1 and cholecystokinin responses were attenuated when compared to liquid untreated caseinate and/or whey protein test products [20]. In other studies the modification of the form of the test foods has also been reflected in postprandial metabolic and appetite responses [1,26-28], also with foods containing only protein [6,29].

In this study, the protein content of the test products was lowered to 6%, which resulted in a less pronounced change in rheological properties of the test product upon crosslinking as compared to our previous study [20]. The crosslinking-induced changes in the structure and viscosity of the test product were not marked enough to induce significant differences in the postprandial responses. In other studies with various model foods, differences in postprandial responses have been observed among the test products in which the modification process has been directed exclusively to the molecular level of the food components without remarkable changes in the physical form of the test food [2-4,30]. These inconsistent results emphasize the importance of better understanding the critical physiology- and food-related aspects that modify postprandial GI functions, such as the rate and extent of GE, digestion and absorption processes.

Hypothetically, TG-induced crosslinking of casein could influence the digestive process in the upper GI tract through changes in its functional characteristics, such as aggregation or gelation behaviour at the low pH-values prevailing in the stomach, which may then affect GE rate and subsequent postprandial glucose, insulin and amino acid responses. Furthermore, altered physicochemical characteristic of the test products could also be reflected in gastric function and appetite sensations [31,32]. However, in the present study the TG-treatment induced no significant effect on postingestive glucose, insulin, AA or appetite responses. This may be because of a low degree of crosslinking and/or low protein concentration in the test beverage, which nonetheless was close to the protein concentration found in regular milk. Fox et al. [33] used a comparable approach investigating the effect of dephosphorylation of casein on gastric emptying. Dephosphorylation was found to reduce the size of aggregates formed under acid conditions and also to increase the rate and extent of pepsin digestion of casein *in vitro*[34]. Such effects would be expected to increase the rate of gastric emptying, but gastric emptying tended to be faster for the unmodified than for the dephosphorylated protein [33].

We included whey protein also in the study protocol to verify the differences between the two main milk protein types, casein and whey protein. The most apparent differences were detected in postprandial concentrations of some plasma AAs, whereas glucose, insulin and appetite responses and subsequent energy intake were similar after these protein types. Several previous studies indicate that casein and whey protein differ in their postprandial responses, especially in terms of GE rate [23], insulin secretion [15,24], AA concentration [14,23,35], and appetite responses [23,25]. However, there are also earlier data supporting the results of the present study showing that insulin [14,20,23,36-38] and appetite responses [20] may not be affected by the different milk protein types with protein-specific AA content. Surprisingly, the results of a recent study by Acheson et al. [39] showed that casein is more satiating than whey and GE is faster after casein compared with whey protein, results that partly challenge the findings of previous studies [23,25]. These inconsistencies in the results on casein and whey proteins in the short-term studies point out the importance of the modulating effects of test product -related factors, e.g. the dosage, concentration and type of single protein used and presence or absence and source of other macronutrients used in the test products.

Comparable insulin profiles among our test products were observed, despite the more pronounced postprandial branched-chain amino acid (BCAA) concentrations after Wh, which have been show to stimulate insulin secretion [39]. We used glucose also in our test products which certainly affected our insulin profiles to a higher extent than the BCAA. In studies where either lactose or no additional carbohydrate was used in the test meals different insulin responses between whey protein and casein were detected [15,24], but not always [36,37]. Furthermore, Acheson et al. [39] who used dextrose in their test products observed a more pronounced postprandial insulin response after whey protein compared to casein. However, unlike in our study the protein/carbohydrate ratio in their study emphasized the protein content which may have also affected the results. Therefore, the additional glucose and the protein/glucose ratio in our study resulted in pronounced postprandial insulinemia, which masked the insulin-stimulating effects of insulinotropic AAs. The total plasma AA concentration is also important for the insulin response as suggested by Nilsson et al. [40] who showed that beverages that caused the highest total postprandial AA increments caused also the highest insulin responses. The total postprandial AA response in our study was comparable among test products as was the insulin response. Furthermore, the form of test product [20] is adding to the complexity of the metabolic system controlling overall homeostasis.

The trend for a faster GE after Wh than after Cas and the differences in certain AA concentrations, especially in the BCAA concentrations between Wh and Cas have been demonstrated previously [23,24,35,37]. Our results indicate that the milk proteins specifically modulate the GE function. In addition to the potential effects of GE rate on AA response, the differences in AA compositions between the whey protein isolate and casein protein may explain the differences in the postprandial plasma AA responses, since milk protein-specific AA composition appears to be reflected in the postprandial AA concentration [41]. Furthermore, the type of casein (micellar casein, sodium/calcium caseinate) is likely to add variation to the overall postprandial AA response profile due to their potentially different postingestive behaviour in the GI tract [42].

## Conclusions

Our study indicates that crosslinking with TG does not alter postprandial metabolism and appetitive responses of sodium caseinate when consumed in the form of a pourable test products, even if crosslinking causes changes in protein molecular structure and a slight increase in the viscosity of the beverage. The changes brought about by crosslinking were probably not extensive enough to result in changes in postprandial gastric functions or protein digestibility.

## Abbreviations

AA, Amino acid; Cas-TG, Caseinate crosslinked by transglutaminase; Cas, Caseinate; GE, Gastric emptying; GI, Gastrointestinal; TG, Transglutaminase; VAS, Visual analogue scale; Wh, Whey protein.

## Competing interests

The authors declare that they have no competing interests.

## Authors' contributions

KRJ, LJK, ML, KHH, DL, LN, HM and KSP designed the research. KRJ conducted the research. ML provided the ingredients and the protocol for preparing the test products. KRJ analyzed the data and wrote the first version of the manuscript. All authors contributed to the writing of the final manuscript and approved it.
